# Stem cells in the treatment of renal fibrosis: a review of preclinical and clinical studies of renal fibrosis pathogenesis

**DOI:** 10.1186/s13287-021-02391-w

**Published:** 2021-06-10

**Authors:** Yiping Liu, Yan-Yan Su, Qian Yang, Tianbiao Zhou

**Affiliations:** 1 Department of Nephrology, the Second Affiliated Hospital of Shantou University Medical College, No. 69 Dongsha Road, Shantou, 515041 China; 2 Department of Nephrology, Huadu District People’s Hospital of Guangzhou, Southern Medical University, Guangzhou, China

**Keywords:** Stem cells, Renal fibrosis, Signaling pathway, Macrophages

## Abstract

Renal fibrosis commonly leads to glomerulosclerosis and renal interstitial fibrosis and the main pathological basis involves tubular atrophy and the abnormal increase and excessive deposition of extracellular matrix (ECM). Renal fibrosis can progress to chronic kidney disease. Stem cells have multilineage differentiation potential under appropriate conditions and are easy to obtain. At present, there have been some studies showing that stem cells can alleviate the accumulation of ECM and renal fibrosis. However, the sources of stem cells and the types of renal fibrosis or renal fibrosis models used in these studies have differed. In this review, we summarize the pathogenesis (including signaling pathways) of renal fibrosis, and the effect of stem cell therapy on renal fibrosis as described in preclinical and clinical studies. We found that stem cells from various sources have certain effects on improving renal function and alleviating renal fibrosis. However, additional clinical studies should be conducted to confirm this conclusion in the future.

## Introduction

Chronic kidney disease (CKD) is a major epidemiological, clinical, and biomedical challenge that has a high prevalence and high mortality. CKD can progress to end-stage renal disease and result in serious economic and social burdens [1]. The main causes of CKD are diabetic nephropathy, hypertensive nephropathy, primary chronic glomerulonephritis, chronic interstitial glomerulonephritis, and chronic tubular disease, and these diseases can induce renal structural changes and dysfunction. Chronic inflammation can stimulate renal fibrosis and is also an important predisposing and progressive factor for CKD [2, 3], and among various cells, macrophages are involved in modulating renal fibrosis in CKD patients [4–6].

Renal fibrosis commonly leads to glomerulosclerosis and renal interstitial fibrosis and its main pathological basis involves tubular atrophy and the abnormal increase and excessive deposition of extracellular matrix (ECM) [7]. Inflammatory cell infiltration, fibroblast activation and expansion, ECM component deposition, tubular atrophy, and microvascular thinning are the main pathological events of renal fibrosis [8]. Compounds that can improve renal progenitor commitment to regenerative may alleviate renal fibrosis and there is convincing evidence indicating that certain compounds can modulate renal tissue with intrinsic regenerative potential. The alleviation of fibrosis alone is not sufficient to repair kidney function in the absence of restoring lost nephron tissue after damage. Consequently, stimulating endogenous tissue regeneration might represent an attractive strategy to treat renal disorders [8, 9]. Current evidence indicates that complement activity transcends innate host defense, and the complement system regulates processes such as the differentiation of stem cells, repair of tissue, and progression to fibrosis.

Stem cells have multilineage differentiation ability and regenerative potential under appropriate conditions and are easy to obtain. At present, there are some studies showing that stem cells can alleviate the accumulation of ECM and renal fibrosis. Stem cells can be divided into two types based on the developmental stage: embryonic stem cells and adult stem cells. Depending on their differentiation potential, stem cells can also be divided into totipotent stem cells, pluripotent stem cells, and monogenic stem cells, which are characterized by multidirectional differentiation, and infinite division and proliferation. Currently, the majority of stem cells used to treat renal fibrosis are mesenchymal stem cells (MSCs), which include bone marrow mesenchymal stem cells (BM-MSCs), umbilical cord blood mesenchymal stem cells (UC-MSCs), amniotic fluid mesenchymal stem cells (AF-MSCs), adipose mesenchymal stem cells (AMSCs), Wharton’s jelly-derived MSCs (WJ-MSCs), and dental mesenchymal stem cells (DMSCs).

**Fig. 1:**
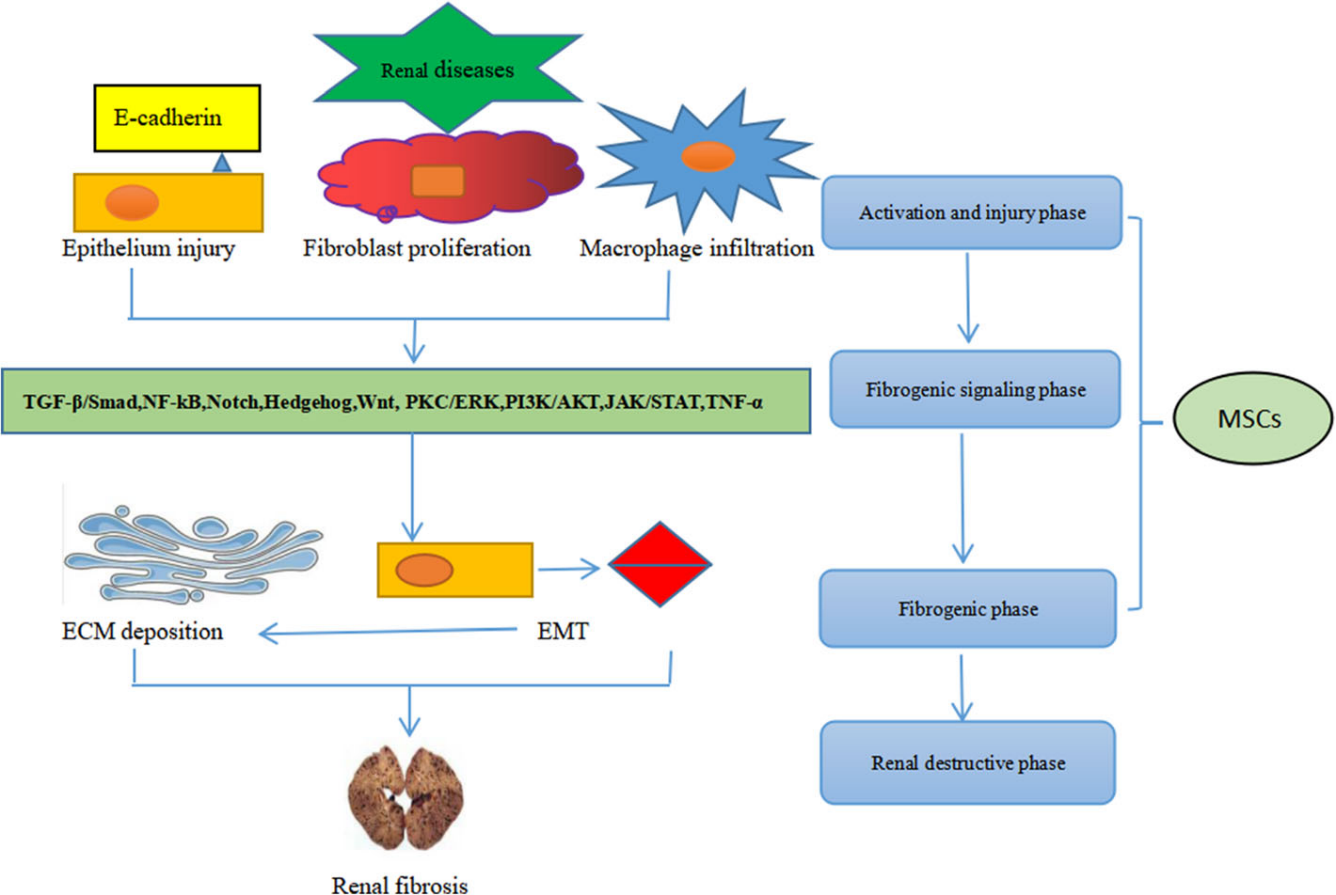
Association of stem cells with renal fibrosis

One of the hallmarks of renal fibrosis is excessive ECM deposition, and ECM accumulation can lead to renal failure. Thus, an imbalance between ECM overproduction and reduction can lead to glomerulosclerosis and tubulointerstitial fibrosis. Injury of podocytes and endothelial cells and mesangial cell proliferation are involved in the pathogenesis of glomerulosclerosis [10]. Several signaling pathways are involved in renal fibrosis, including nuclear factor-κB (NF-κB) [11], transforming growth factor-β1 (TGF-β1)/Smad [12], Notch, Wnt, Hedgehog [13], phosphatidylinositol-3 kinase (PI3K/AKT), transcription/signal transducers and activators of transcription (JAK-STAT), RHO/Rho coil kinase (ROCK), and tumor necrosis factor α (TNF-α). However, among these pathways, the TGF-β1/Smad signaling pathway is considered the central pathway that mediates the progression of renal fibrosis and chronic renal disease, and the TGF-β1/Smad signaling pathway is extensively associated with other signaling pathways during fibrosis [14]. Renal epithelial cell damage can be caused by ischemia and toxins, induces proteinuria in many diseases, such as glomerulonephritis, diabetes nephropathy, or hypertension nephropathy, and may result in fibroblast proliferation and macrophage infiltration [15, 16]. TGF-β1 released from damaged and infiltrated cells and acts on kidney fibroblasts [17], subsequently causing epithelial to mesenchymal transformation (EMT) and abnormal ECM deposition. Furthermore, fibrosis-promoting molecules, including collagen, fibronectin, and plasminogen activator inhibitor-1 (PAI-1), may contribute to renal failure. Stem cells can delay the progression of renal fibrosis. MSCs can influence the activation of proinflammatory cytokines and inhibit fibrosis signaling pathways, mainly TGF-β1/Smad, NF-κB, mitogen-activated protein kinase (MAPK)/ERK, and PI3K/AKT [18–22]. In addition, AMSCs can reduce IL-1β, TNF-α, and IL-6 during the treatment of renal interstitial fibrosis and inhibit activation of the TGF-β1/Smad2/3/7 signaling pathway [23]. MSCs can inhibit fibrosis by reducing EMT and can ameliorate renal fibrosis by decreasing ECM accumulation (Fig. 1).

However, the sources of stem cells and the types of renal fibrosis or renal fibrosis models used in these studies are varied. In this review, we summarize the pathogenesis (including signaling pathways) of renal fibrosis and the effect of stem cell therapy on renal fibrosis in preclinical and clinical studies.

## Signaling pathway in renal fibrosis

### The TGF-β/Smad signaling pathway

Tissue fibrosis is a common characteristic of chronic diseases that lead to organ failure. The main cell type that contributes to the increase in collagen deposition in active tissue fibrosis is the myofibroblast-α subgroup of activated fibroblasts, which are characterized by the expression of α-smooth muscle actin (α-SMA) [24, 25]. Myofibroblasts are a heterogeneous population that may originate from more than one precursor population [26] and can arise through processes including EMT [27, 28], endothelial-mesenchymal transformation (EndoMT) [29–31], and the local proliferation of resident fibroblasts or pericytes. The study by Wang et al. [32] showed that macrophage-myofibroblast transition (MMT) is the main source of myofibroblasts. This process occurs in the fibrotic kidney and is regulated by the TGF-β/Smad3 signaling pathway [33].

There are four main mechanisms by which TGF-β1 promotes fibrosis.

1. TGF-β1 increases the generation of ECM components (type I collagen and fibronectin) through a Smad3-dependent or Smad3-independent mechanism [34–36].
2. TGF-β1 suppresses ECM degradation by suppressing matrix metalloproteinases (MMPs) [37–39]. Tissue inhibitors of metalloproteinases (TIMPs) are natural inhibitors of MMPs [40].
3. TGF-β1 is believed to play an important role in the transformation of epithelial cells, endothelial cells, and pericytes into myofibroblasts [25, 41].
4. TGF-β1 can immediately act on different types of resident renal cells and promote the proliferation of glomerular mesangial cells, increase matrix production, or decrease the disappearance of renal tubular epithelial cells and podocytes, thus exacerbating renal injury and leading to worsened renal fibrosis [42, 43].

**Fig. 2:**
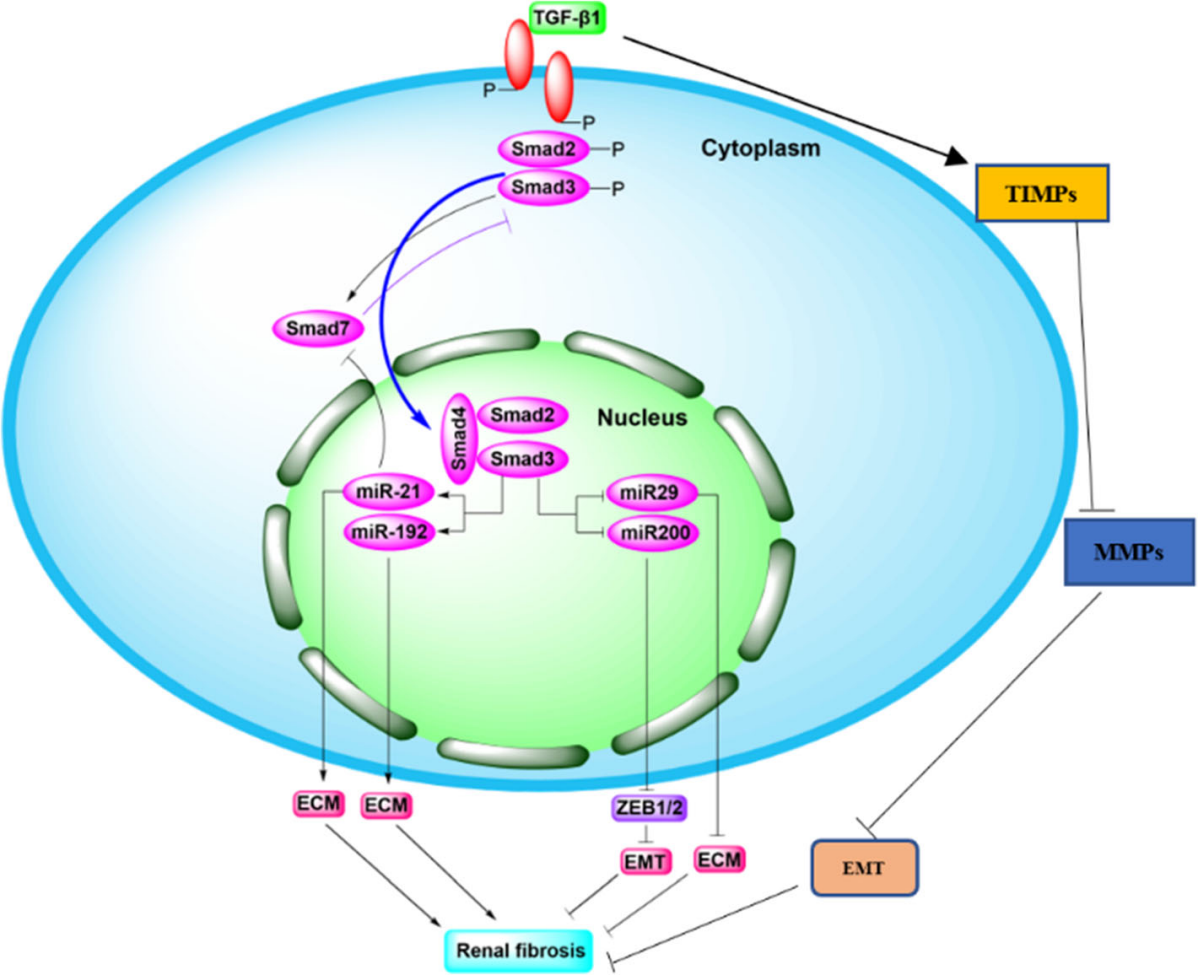
TGF-β/Smad signaling pathway in fibrotic kidney

TGF-β1 acts through TGF-β receptors and Smad2/3 transcription factors [42, 44, 45]. Smad3 can directly bind to the promoter region of the collagen gene to trigger collagen production, induce TIMP-1 to reduce the activity of MMP-1 in fibroblasts, inhibit the degradation of ECM, and promote fibrosis due to various causes [46–48], while some studies have shown that the role of smad2 in renal fibrosis is the opposite of that of smad3 [49–51]. During fibrogenesis, the downregulation of Smad7 is inhibited by a ubiquitin E3 ligase-dependent degradation mechanism, and the shift in balance between Smad3 and Smad7 leads to myofibroblast accumulation and activation, ECM overproduction, and reduced ECM degradation. Moreover, current studies have shown that Smad3 mediates renal fibrosis by downregulating miR-29 and miR-200, and upregulating miR-21 and miR-192. MiR-21 and miR192 accelerate ECM deposition, which in turn promotes renal fibrosis [52, 53]. The overexpression of miR-29 reduces the degree of renal fibrosis in vivo and inhibits fibrotic genes in vitro in the context of ECM deposition-associated diabetic nephropathy [53–55]. MiR-200 can inhibit TGF-β1-induced EMT in peritoneal mesothelial cells by regulating ZEB1/2 [56] (Figs. 2 and 6).

### TGF-β/Smad-independent signaling pathways in renal fibrosis

#### The Notch signaling pathway in renal fibrosis

In recent years, Notch was confirmed to be directly involved in renal fibrosis in a gene knockout mouse model [57–59]. In vitro, Notch signaling is directly involved in the EMT process [60, 61] by interacting with TGF-β/Smad2/3 signaling or directly regulating key EMT molecules, such as Snail and Slug [62, 63]. In addition, the Notch signaling pathway can activate the expression of TGF-β in glomerular mesangial cells stimulated by high glucose, suggesting that Notch can participate in the development of fibrosis by regulating key factors of fibrosis. Blocking the Notch signaling pathway reduced the degree of renal fibrosis in a model of unilateral ureteral obstruction (UUO)-induced renal fibrosis, and this effect was achieved by inhibiting the activation of the TGF-β/Smad signaling pathway [64]. In addition, a study also showed that blocking the Notch signaling pathway in renal endothelial cells in the context of renal EMT inhibited the occurrence of arteriovenous fistula renal failure [65], suggesting that Notch may also play an important role in renal EMT. Furthermore, the Notch signaling pathway regulates the involvement of macrophages in renal fibrosis.

**Fig. 3:**
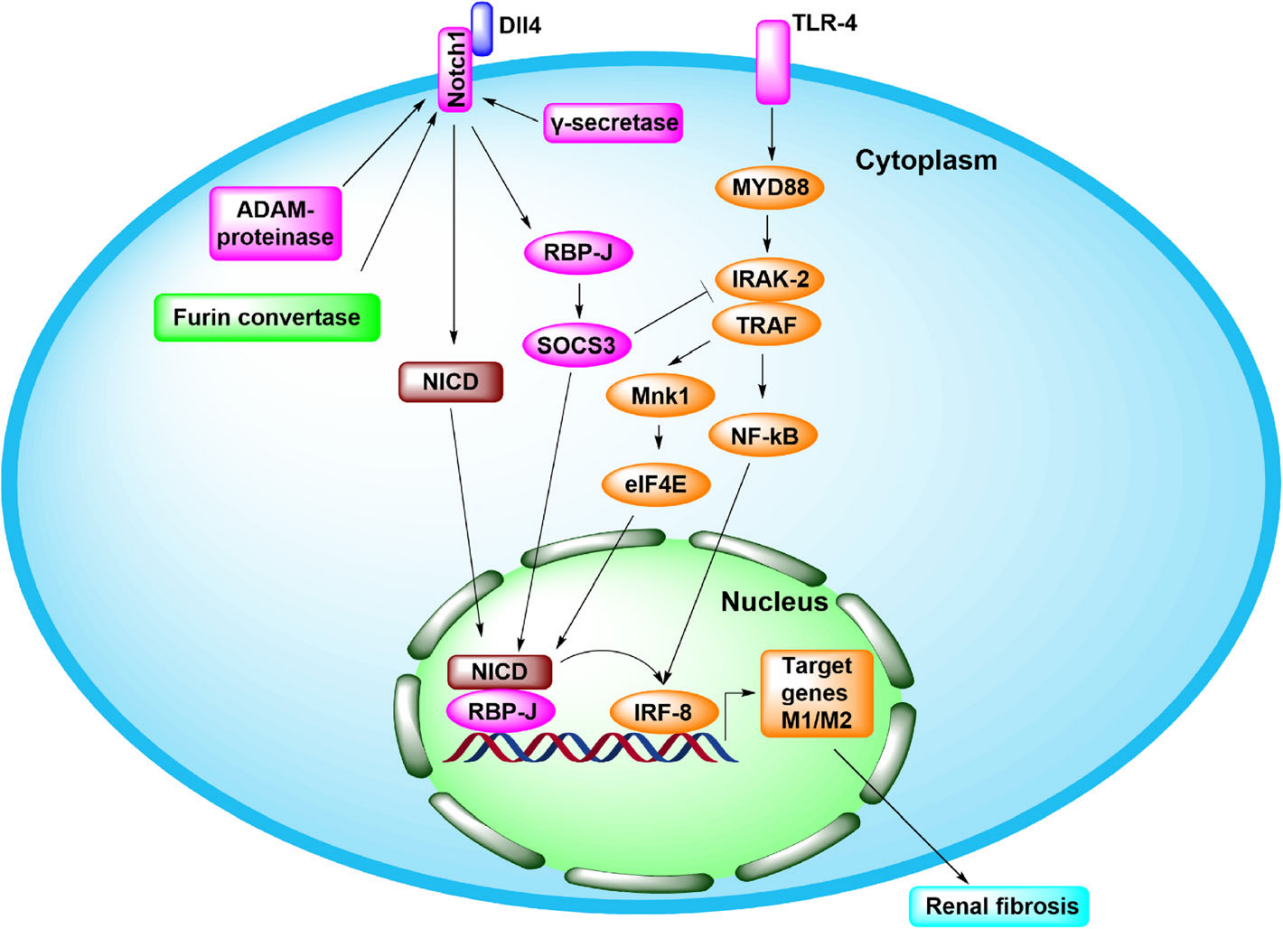
Notch signal pathway in fibrotic kidney

There are 29-36 EGF repeats and 3 Lin-Notch repeats in the extracellular domain of the Notch receptor, which can bind with ligands to activate the signaling pathway. There are three cleavage sites (S1, S2, S3 and S4) in the transmembrane region, which can be cleaved by furin convertase, ADAM-protease, and γ-secretase to produce the Notch intracellular domain (NICD) [66]. TLR4 participates in the activation of Notch signaling by regulating the IRAK2-Mnk1-eIF4E axis and NF-κB activation, and the overexpression of downstream molecules can in turn inhibit Notch-mediated regulation of macrophage activation [67]. Wang et al. [32] showed that bone marrow-derived M1 macrophages inhibited tumor growth under tumor-bearing conditions, while M2 macrophages had the opposite effect, and M1 macrophages were susceptible to Notch signaling regulation. Notch signaling participates in the activation of M1/M2 macrophage by regulating SOCS3 [68, 69]. The Notch-RBP-J signaling pathway can also promote the activation of inflammatory M1 macrophages by regulating IRF8, and regulatory mechanism is mediated by the TLR4-IRAK2-Mnk1-eIF4E signaling pathway [67] (Figs. 3 and 6).

#### The Hedgehog signaling pathway in renal fibrosis

In the development of fibrosis, Hedgehog (Hh) genes in mammals can be divided into three categories: Sonic hedgehog (Shh), Desert hedgehog (Dhh), and Indian hedgehog (Ihh), which encode the proteins Shh, Ihh, and Dhh, respectively [70]. Shh is the most widely expressed type and mainly plays a role in the signaling pathway; Ihh mainly participates in the proliferation and differentiation of bone cells; and Dhh participates in the development of reproductive cells. The Shh protein is composed of the receptor transmembrane protein patched homolog (PTCH), its downstream proteins Smoothened (Smo) and Zinc finger protein (Gli). During embryonic development, the Shh protein signals through the Hedgehog signaling pathway [71, 72]. Hedgehog signaling pathway proteins are expressed in the early stage of renal injury and participate in tissue repair after renal injury. Renal interstitial cells induced by the Shh gene are closely associated with renal fibrosis. The Shh signaling pathway affects the transformation of not only fibroblasts to myofibroblasts, but also pericytes to myofibroblasts. Fabian et al. [73] showed that Shh ligands trigger cell proliferation and induce pericytes to regulate myofibroblasts, and the cell precursors are transformed into myofibroblasts.

**Fig. 4:**
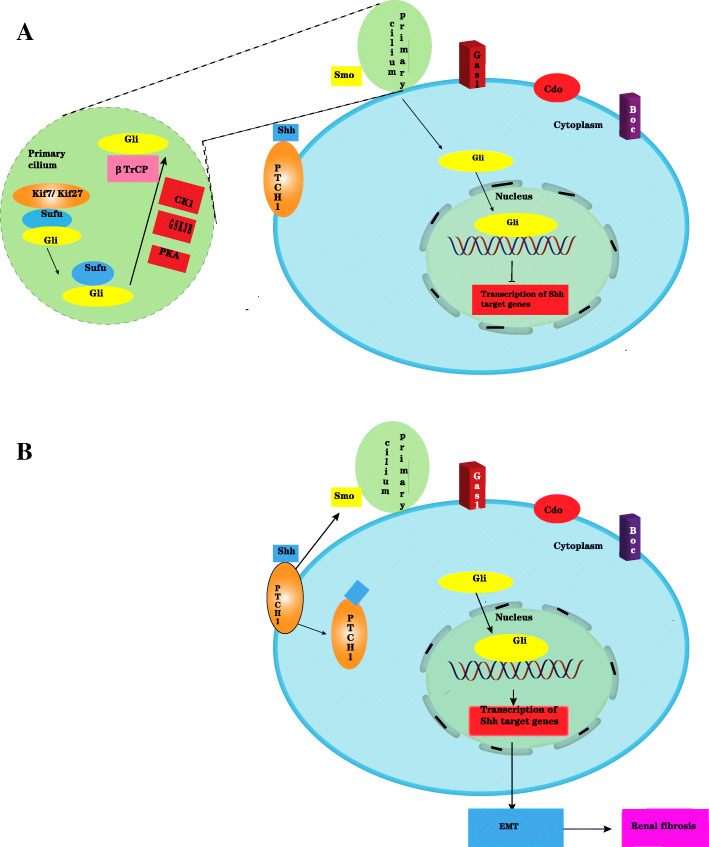
Hedgehog signal pathway in healthy kidney (**a**) and fibrotic kidney (**b**)

Due to the lack of Hh molecules, Gli protein homeostasis is disrupted by KiF7/KiF27 and fusion inhibitors (Sufu), followed by the phosphorylation of the Gli, Smo, and Sufu complex by protein kinase A (PKA), glycogen synthase kinase 3B (GSK3B), and casein kinase 1 (CK1). The transduction protein repeat-containing protein βTrCP cleaves Gli, and the C terminus of Gli is shortened. The C-terminal shortened Gli repressor enters the nucleus and binds to DNA to inhibit the transcription of Shh target genes. The Hh molecule is present in the fibrotic kidney, where the Shh molecule binds to the receptor Patched1 (PTCH1), and Ptc loses its inhibition of Smo. Phosphorylated Sufu loses its obstruction to Gli, and Gli enters the nucleus and binds to DNA to activate Hh target genes. The activated Hh target genes can promote EMT. Thus, the Hedgehog signaling pathway can promote renal fibrosis [74] (Figs. 4 and 6).

#### The Wnt signaling pathway in renal fibrosis

**Fig. 5:**
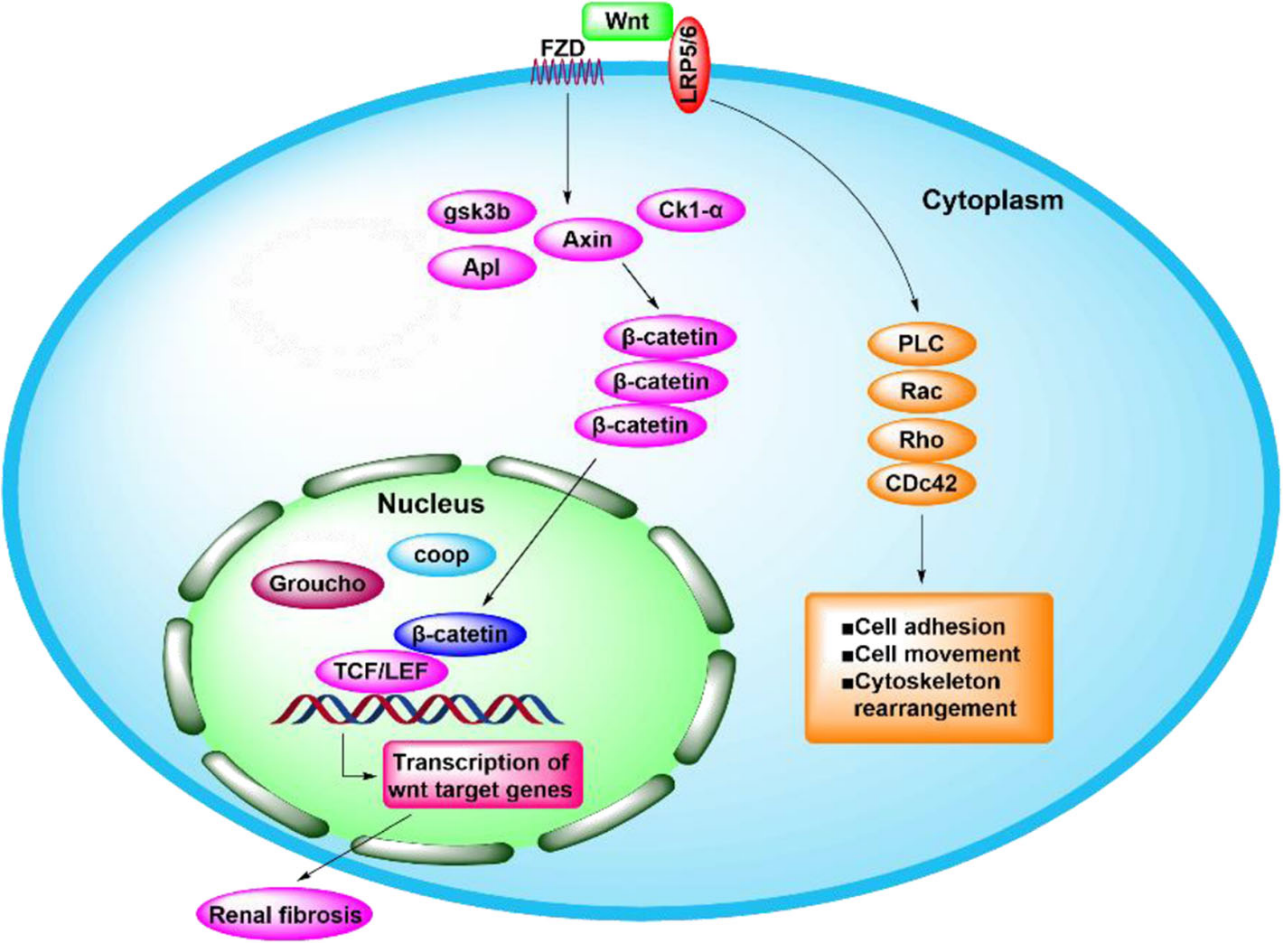
Wnt signal pathway in fibrotic kidney

**Fig. 6:**
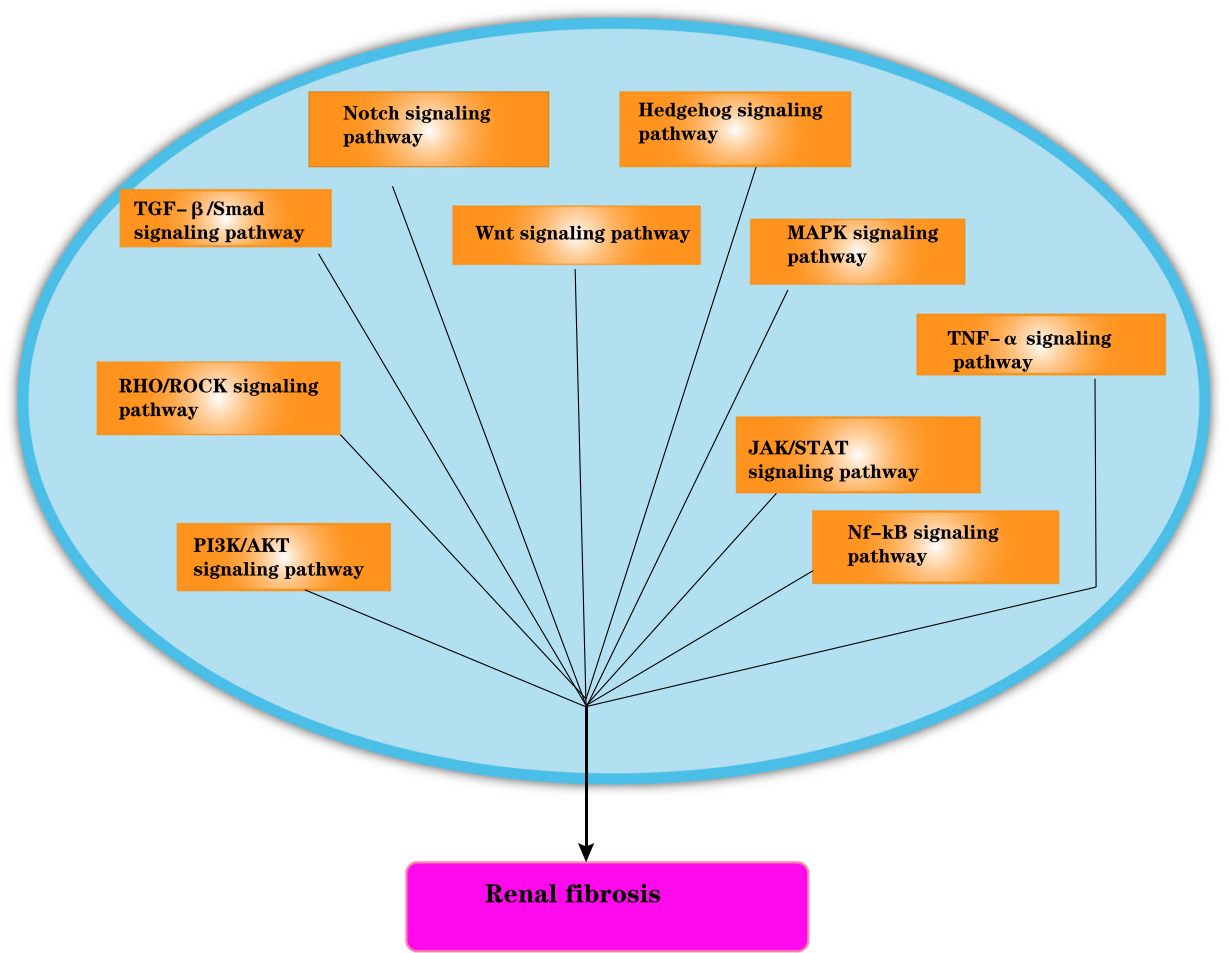
The potential signaling pathways for renal fibrosis

The Wnt signaling pathway plays an important role in tumorigenesis, and this pathway is also important in embryonic development [75, 76] and stem cell differentiation [73, 77]. In the UUO model of renal fibrosis, the majority of Wnt proteins (except Wnt5b, Wnt8b, and Wnt9b) and 10 Fzd receptors (except Fzd4 and Fzd5) were upregulated [78]. Mechanistically, Wnt and β-catenin participate in the development of fibrosis. Due to the lack of Wnt ligands in fibrotic kidneys, β-catenin in the cytoplasm is phosphorylated by a “destructive complex” containing GSK-3b, the scaffold protein Axin, and casein kinase 1a (CK1a) and is then cleaved by ubiquitin-mediated proteasome. The cytoplasmic translocation of phosphorylated β-catenin leads to the accumulation of noncytoplasmic signals, which allows β-catenin to enter the nucleus, bind with TCF/LEF, and activate Wnt target genes [13, 73] (Figs. 5 and 6).

#### The RHO/ROCK signaling pathway in renal fibrosis

Rho coil kinase (rock) is an important downstream effector of GTPase-RHOA and includes Rock1 (also known as p160 rock and rock β) and Rock2 (also known as Rho kinase and rock α). ROCK genes are located on chromosome 18 (18q11.1) and chromosome 2 (2p24). Rocks have a vital effect on the formation of actin myosin contractility and the regulation of actin cytoskeleton dynamics. These factors play important roles in regulating various cell functions, such as growth, migration, apoptosis, metabolic regulation of cytoskeletal actin synthesis, and cell contraction through the phosphorylation of numerous downstream targets [79]. Activation of RHOA stimulates Rho-related curl protein kinase (rock), a downstream effector, to phosphorylate LIMK, MLC, and MLCP, thus affecting many cellular processes [80]. LIMK is phosphorylated and induced to phosphorylate cofilin (an actin-binding protein). Cofilin plays an important role in the dynamic regulation of actin, leading to depletion of the G-actin pool, promoting actin polymerization (F-actin), and resulting in stress fiber formation, gene expression, and cell transformation (fibroblast to myofibroblast, EMT) [81]. EMT is a crucial step in the development of renal fibrosis and may be involved in the regulation of renal fibrosis, but the specific mechanism remains to be further studied (Fig. 6).

#### The JAK/STAT signaling pathway in renal fibrosis

The JAK/STAT pathway is involved in pleiotropic growth and cytokine cascade signal transduction. At present, the JAK/STAT signal transduction model shows that JAK is activated by the binding of cytokines or growth hormone to receptors, which phosphorylates the intracellular domain of the receptor and allows for the recruitment and phosphorylation of STAT [82]. The STAT3 activation is associated with tissue fibrosis and improves TGF-β1 production. The results of animal model studies [83, 84] showed that STAT3 mediated renal fibrosis by inhibiting leukocyte infiltration and proinflammatory cytokine expression and the activation of renal interstitial fibrosis. The JAK/STAT signaling pathway may be involved in the regulation of renal fibrosis, but the specific mechanism remains to be further studied (Fig. 6).

#### The PI3K/AKT signaling pathway in renal fibrosis

The PI3K/AKT pathway plays a significant role in cell growth, survival, and metabolism. PI3K is one of the key pathways in the phosphorylation of Ptdlns (3,4,5) P3 and AKT. Phosphatase and tensin homolog (PTEN) deletion from the chromosome 10 gene is a phosphatidylinositol 3'-phosphatidylinositol enzyme that can convert Ptdlns (3,4,5) P3 into phosphatidylinositol (4,5)-diphosphate. For a long time, PTEN was considered to have antiphosphatase activity against phosphatidylinositol PIP3 and phosphatidylinositol PI3Ks [85]. Some studies have indicated that PI3K/AKT has an important regulatory effect on organ fibrosis, including pulmonary fibrosis [86], myocardial fibrosis, renal fibrosis [87], and liver fibrosis [88]. Recently, a study [89] indicated that flunidone inhibits nicotinamide adenine dinucleotide phosphate oxidase through the PI3K/AKT signaling pathway during the pathogenesis of renal interstitial fibrosis, indicating that the PI3K/AKT signaling pathway is involved in the EMT process of renal interstitial fibrosis. The function of PI3K/AKT is to catalyze intracellular phosphatidylinositol triphosphate formation and the phosphorylation of AKT, which is one of the key downstream pathways of PI3K. PI3K/AKT signaling is involved in EMT fibrosis in the intercellular matrix. In particular, the PI3K/AKT/mTOR signaling pathway and many upstream and downstream factors play important regulatory roles in renal fibrosis [90]. Moreover, PI3K may be modulated by TGF-β1 and play biological roles [91]. PI3K/AKT may participate in the regulation of renal fibrosis, but the concrete mechanism remains to be further studied (Fig. 6).

#### TNF-α in renal fibrosis

EMT plays an important role in the pathogenesis of renal fibrosis, and TNF-α can cooperate with TGF-β to regulate EMT [92]. It has been proven that TNF-α can significantly enhance TGF-β-mediated promotion of EMT. In a study of the human adenocarcinoma epithelial cell line A549, TNF-α promoted TGF-β-induced EMT, induced an interstitial cell phenotype, upregulated expression of the interstitial cell markers α-SMA and type I collagen, downregulated expression of the epithelial marker E-cadherin, and increased cell invasiveness and matrix metalloproteinase secretion. The possible mechanism involved enhancing the effects of the Smad, MAPK, and NF-κB signaling pathways on EMT [93]. TNF-α may be involved in the regulation of renal fibrosis, but the specific mechanism remains to be further studied (Fig. 6).

#### The MAPK signaling pathway in renal fibrosis

The main subsets of MAPK signaling pathways are ERK, P38 kinase, and JNK. The MAPK and TGF-β signaling pathways jointly promote the progression of EMT [94]. These transcription factors can inhibit E-cadherin and induce the expression of TGF-β receptors. The MAPK and TGF-β signaling pathways jointly promote the progression of EMT [95]. In addition, studies have shown that the composition of snail and CREB-binding protein (CBP) can stimulate the inflammatory response to renal fibrosis [96] (Fig. 6).

#### NF-κB in renal fibrosis

NF-κB is one of the main nuclear transcription factors that regulates inflammation and the immune response. In the cytoplasm, NF-κB is inactive due to its binding to inhibitory proteins (IKKβ, IKKα, and IKKγ). When the body is stimulated, IKKα is phosphorylated by protein phosphorylase, and phosphorylated IKKα is degraded by protein kinase and dissociates from the NF-κB dimer. NF-κB is activated, exposing p50 and p65, and translocates to the nucleus to bind with the corresponding target gene κB sequence to induce and enhance the expression of EMT-related proteins. Huber and others confirmed that inhibiting NF-κB signaling in Ras-transfected epithelial cells could prevent the occurrence of EMT, and activation of this pathway could promote the morphological transformation of cells to mesenchymal cells in the absence of TGF-β. In addition, the inhibition of NF-κB activity in mesenchymal cells can lead to a reversal of the EMT process, suggesting that NF-κB is necessary for the induction and maintenance of EMT [97]. NF-κB may be involved in the regulation of renal fibrosis, but the specific mechanism remains to be further studied (Fig. 6).

## Macrophages and renal fibrosis

Current evidence shows that macrophage activation can induce various signaling pathways and promote fibrosis. In the UUO model, macrophage infiltration was obvious at 4 h after injury, peaked at 24 h, and was maintained at approximately 10 times higher than normal levels 3 days later [98]. Macrophages are divided into classically activated M1 macrophages and activated M2 macrophages based on the activation mechanisms or cellular functions [99]. M1 macrophages are stimulated by lipopolysaccharide (LPS), interferon-γ (IFN-γ), and TNF-α. M1 macrophages are involved in the initial stage of inflammation. M2 macrophages are activated by IL-4/10/13, TGF-β, and sphingosine 1-phosphate (SP1). M2 macrophages produce growth factors, anti-inflammatory factors, and proangiogenic cytokines, and M2 macrophages are involved in the wound healing process (repair stage). It has been confirmed that the release of MCP-1 and other chemokines caused by high levels of macrophage infiltration after kidney injury [100]. Infiltrating macrophages can further directly cause apoptosis and tissue damage through the production of reactive oxygen species (ROS) and the expression of a variety of cathepsins. Infiltrating macrophages can also decrease apoptosis through the expression of inflammatory factors such as TNF-α, IL-1/6/12, and CS2/3/4. Additionally, macrophages can produce many chemokines and proinflammatory factors and further recruit other innate and acquired immune cells to the injured tissue to promote tissue damage. The characteristic feature of macrophage activation is heterogeneity. In recent years, macrophages have been shown to not only produce proinflammatory factors to exacerbate tissue damage and promote the progression of fibrosis but also accelerate the progression of fibrosis by directly producing profibrosis factors [101–103]. In addition, macrophages can produce IL-10 and TGF-β after the phagocytosis of apoptotic cells, and these factors can induce the proliferation of myofibroblasts and promote the occurrence of renal fibrosis [104, 105]. However, current studies have shown that M2 macrophages are the main infiltrating macrophages in the kidney on the 10th day of UUO, and these M2 macrophages promote the progression of late fibrosis by releasing a large amount of TGF-β [106, 107].

**Fig. 7:**
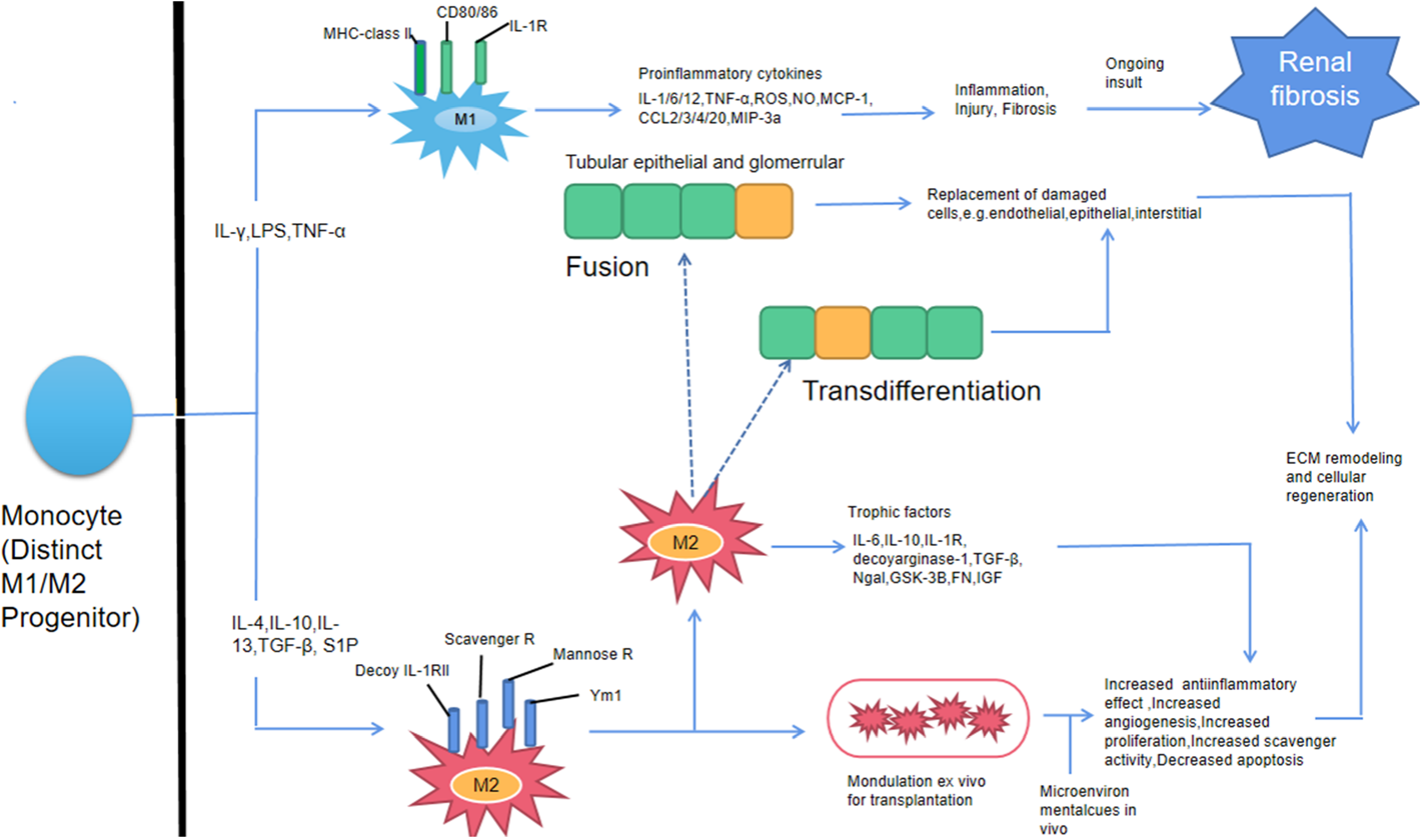
Macrophages and renal fibrosis

In summary, macrophages in renal fibrosis can be classified into three types: proinflammatory macrophages, fibrogenic macrophages, and fibrinolytic macrophages. These findings not only provide new clues for understanding the mechanism of the occurrence and development of renal fibrosis but also indicate that macrophages may be a new target for the treatment of renal fibrosis (Fig. 7).

## Preclinical studies of stem cell therapy for renal fibrosis

### Therapeutic effects of BM-MSCs on renal fibrosis

In 1966, BM-MSCs were first identified by Friedenstein et al. [108]. Da Silva et al. [109] found that BM-MSCs and their conditioned medium (CM) could reduce the production of collagen 1, TNF-α, α-SMA, proliferating cell nuclear antigen (PCNA), and caspase 3 to impede EMT in a UUO rat model. Furthermore, renal arterial injection of BM-MSCs also reduced EMT in a UUO rat model. Asanuma et al. [110] injected MSCs through the renal artery in a UUO rat model and showed that MSCs could decrease α-SMA, FSP+ cell, TNF-α, and total collagen levels, EMT and renal fibrosis; and increase E-cadherin. However, EVGF, TGF-β1, IL-10, FGF, and hepatocyte growth factor (HGF) were not changed. The results of animal experiments by Lang and Dai [111] showed that BM-MSCs could significantly alleviate renal fibrosis in a diabetic nephropathy rat model, and the mechanism may be associated with the inhibition of the TGF-β1/Smad3 pathway, decreased PAI-1 protein expression, reduced ECM accumulation, and the balance of the fibrinolytic system. BM-MSCs can also decrease urinary protein, and Scr. Wu et al. [112] showed that after stem cell treatment, the level of BUN decreased, tubular CCL-2 and CCL-5 decreased, and α-SMA and type IV collagen accumulation significantly decreased, and BM-MSCs had anti-inflammatory and antifibrotic effects on renal tubular cells. Milk globule epidermal growth factor 8 (MFG-E8) is a secretory multifunctional glycoprotein that usually exists in human milk globules. Shi et al. [113] showed that BM-MSC-EVs could decrease renal fibrosis by producing MFG-E8, which suppressed the RhoA/ROCK pathway. Ninichuk et al. [114] injected BM-MSCs via the tail vein in COL4A3-deficient mice (Alport disease model) and showed that BM-MSCs could decrease BUN and Scr and inhibit glomerulosclerosis and renal fibrosis, but EVGF and BMP7 were not changed. Wang et al. [115] injected BM-MSCs via the vein into UUO mice and showed that BM-MSCs could decrease Kim-1, Col4a1, TGF-β1, TGF-βR1, α-SMA, and renal fibrosis. Matsui et al. [116] injected BM-MSCs through the renal artery in a UUO rat model, and the results indicated that BM-MSCs could decrease Col-I, Col-III, fibronectin, α-SMA, p-STAT3, MMP-9, TIMP-1, and renal fibrosis and increase TIMP-1/MMP-9 (Table 1).

**Table 1:** Summary of evaluation of the efficacy of stem cells in treating renal fibrosis

Author, year	Stem cell type	Animal model	Groups	Handling methods	Treatment effect
Ninichuk et al. 2006 [114]	BM-MSCs	COL4A3-deficient mouse (Alport disease model)	Wild-type group (n = 6), Collagen4A3−/− + saline group (n = 10), Collagen4A3−/− + MSC group (n = 10)	Injected with BM-MSCs (1 × 10^6^) or vehicle via tail vein	BUN↓, Scr↓, EVGF→, BMP7→, inhibit glomerulosclerosis and renal fibrosis
Asanuma et al. 2011 [110]	AD-MSCs	UUO rat	Sham group; UUO group; UUO + AD-MSCs group	Cells (1 × 10^6^) were injected through the renal artery	α-SMA↓, FSP+ cell ↓, TNF-α↓, total collagen content↓, E-cadherin↑, EVGF→, TGF-β1→, IL-10→, FGF→, HGF→, inhibit EMT and renal fibrosis
Donizetti-Oliveira et al. 2012 [117]	AMSCs	IRI mice	Sham group; IR group; IR + AMSCs group	Cells (2 × 10^5^) were intraperitoneally administered to each mice	IL-1α↓, IL-6 ↓, TNF-α↓, IL-4, IL-10↑, IL-12↓, HO-1↑, RANTES↓, FSP-1↓, Col-I↓, hypoxyprobe↓, KC→, IL-1β→, IL-13→, inhibit renal fibrosis
Du et al. 2012 [22]	WJ-MSCs	IRI rat	(i) Normal rats (n = 8); (ii) sham-operated rats (n = 16); (iii) vehicle-injected IRI rats (n = 16); (iv) WJ-MSC-injected IRI rats (n = 16)	Intravenous infusion of 2 × 10^6^ WJ-MSCs	Scr↓, BUN↓, p-Akt↑, HGF↑, HO-1↑, IL-10↑, collagen↓, α-SMA↓, renal fibrosis↓ renal tubular cell apoptosis↓, renal tubular cell proliferation↑
Sedrakyan et al. 2012 [118]	AF-MSCs	Transgenic Alport C57BL/6 mice (Col4a5 knockout mice)	(1) Wild-type C57BL/6 mice (n = 15), (2) Col4a5−/− mice (n = 25), and (3) Col4a5−/− mice + mouse AFSC	Cells (1 × 10^6^) were injected into the left ventricle	Col4a1↑, Col4a2↑, Col4a3↑, Col4a4↑, Col4a5↑, Col4a6→, glomerular and interstitial fibrosis↓
Du et al. 2013 [119]	WJ-MSCs	IRI rat	(a) Sham group (n = 24); (b) unilateral IRI group (n = 24); (c) unilateral IRI plus MSC-injected group (n = 24); and (d) unilateral IRI plus nephrectomized group (n = 24)	Intravenous infusion of 2 × 10^6^ WJ-MSCs	Total renal collagen concentration↓, E-cadherin↑, α-SMA↓, HGF/TGF↑, renal fibrosis↓
Huang et al. 2013 [120]	uUC-MSCs	UUO rat	UUO group, n = 21; UUO + MSC group, n = 21; Sham group, n = 21; Sham + MSC group, n = 21	Injected with MSCs (5 × 10^6^) or vehicle via tail vein	Inhibit renal fibrosis
Katsuno et al. 2013 [121]	AMSCs	AKI rat model induced by folic acid	Control group, AKI + hHASCs group, and AKI + hLASCs group	Intravenous injection	Scr→, BUN→, HGF→, VEGF→
Quimby et al. 2013 [122]	AMSCs	Cat CKD model	Study 1 (six cats) received 2 × 106 cryopreserved aMSCs per infusion, study 2 (five cats) received 4 × 10^6^ cryopreserved aMSCs per infusion, study 3 (five cats) received 4 × 10^6^ aMSCs	4 × 10^6^ MSCs intravenously × 3 treatments	Scr→, BUN→, IL-8→, MCP-1→, TGF-β1→, VEGF→
Sun et al. 2013 [123]	AF-MSCs	UUO mice	Normal group, UUO group, UUO + hAFSCs group	3.5 × 10^5^ MSCs were injected via tail vein	HIF-1α↓, TGF-β1↓, Col-I↓, MCP-1↓, VEGF↑, E-cadherin↑, PCNA↑, Ki67↓, cell apoptosis↓, renal fibrosis↓
Zhou et al. 2013 [124]	uUC-MSC exosome	Cisplatin-induced AKI rat model	Normal group; AKI group; AKI + hucMSC-ex group; AKI+ hucMSC-CM group; AKI + non-hucMSC-ex; group; HFL-1-ex group	200 μg hucMSC-ex was injected into the kidneys via the renal capsule	8-OHdG↓, GSH↑, MDA↓, caspase 3↓
Baulier et al. 2014 [125]	AF-MSCs	Kidney transplantation model induced with IRI (pig)	The vehicle group, the AF-MSC group, the control group	Cells (1 × 10^6^) were injected into the renal artery of the grafted kidney	Scr↓, proteinuria↓, α-SMA↓, VEGF-A↓, Ang 1, Flt-1↓, renal fibrosis↓
Iwai et al. 2014 [126]	AMSCs	Rat DCD renal transplantation	Control group, MSC(−) group, MSC(+) group	MSCs were injected systemically via the penile vein or via the renal artery	Scr↓, BUN↓, renal fibrosis↓, rat survival↑
Wu et al. 2014 [112]	BM-MSCs	Mouse model of protein overload proteinuria	Uninephrectomized (UNX) group, UNX + MSCs group, UNX + BSA group and UNX + BSA + MSCs group	Mouse BM-MSCs (1 × 10^6^ cells/mouse) were injected intravenously into uninephrectomized mice	CCL-2↓, CCL-5↓, α-SMA↓, Col-IV↓
Zhang et al. 2014 [127]	WJ-MSCs-MV	IRI rat	Sham-operated rats (n = 6); vehicle-injected IRI rats (n = 6); MVs-injected IRI rats (n = 6)	100 μg MVs in 1 mL vehicle was administered via caudal vein immediately after reperfusion	Scr↓, BUN↓, NOX2↓, α-SMA↓, MDA↓, ROS↓, Ki67↓, cell apoptosis↓, renal fibrosis↓
Burgos-Silva et al. 2015 [128]	AMSCs	Kidney injury mice induced by folic acid	FA + AMSCs group, FA group, Bic group, Bic + AMSCs group, control group	Via intraperitoneally into FVB mice (1 × 10^6^ cells per animal)	CXCL1↓, CCL-5↓, MPO↓, PCNA↓, MCP-1→, IL-2→, IL-6→, GM-CSF→, MIP-1a→, BUN→
Cunha et al. 2015 [129]	AF-MSCs	IRI rat	Group I/R+ vehicle; group I/R + hAFSC; group no injury	Cells (1 × 10^6^) were injected into the renal artery	Ki67↓, α-SMA↓, CD68↓, Scr→, tubular necrosis↓, tubular hyaline casts↓, renal fibrosis↓
Hattori et al.2015 [130]	DMSCs	IRI mice	SHED group; DMSCs group; control group	Administered injected into the subrenal capsule	Scr↓, BUN↓, MIP-2↓, IL-1β↓, MCP-1↓, macrophages and neutrophils infiltration↓
Lang et al. 2016 [111]	BM-MSCs	DN rat (STZ-induced)	Normal control group (NC group, n = 10), diabetic nephropathy group (DN group, n = 10), stem cell transplantation group (MSC group, n = 10)	2 × 10^6^ BM-MSCs via tail vein	Urinary protein↓, Scr↓, PAI-1↓, TGF-β1↓, Smad3↓, inhibit renal fibrosis
da Silva et al. 2015 [109]	BM-MSCs	UUO rat	Sham, UUO, UUO + BM-MSC, and UUO + CM	1 × 10^6^ BM-MSCs via cava vein	Col-lA1↓, α-SMA↓, TNF-α↓, activated caspase 3↓, PCNA ↓(7 days) ↑(14 days); inhibit renal fibrosis
Wang et al. 2016 [115]	BM-MSCs	UUO mice	Sham group, UUO group, UUO + MSCs group, UUO + miR-let7cMSCs group (n = 5/group/time point)	Cells (1 × 10^6^) were intraperitoneally administered to each mice	Kim-1↓, Col4a1↓, TGF-β1↓, TGF-βR1↓, α-SMA↓, renal fibrosis↓
Zou et al. 2016 [131]	uUC-MSC	IRI rat	Sham group, IRI group, IRI + EVs group and IRI + EVs-RNase group	100 μg MVs in 1 mL vehicle was administered via caudal vein immediately after reperfusion	Scr↓, BUN↓, Ki67↑, cell apoptosis↓, HIF-1α↓, VEGF↑, PHD2↑, VHL↑, α-SMA↓, renal fibrosis↓
Eirin et al. 2017 [132]	AMSCs	RAS pig model	Lean group, MetS group, MetS + RAS group, MetS + RAS + EVs group, MetS + RAS + IL10 KD EVs group	MSCs were injected via the renal artery	Scr↓, macrophages M1↓, macrophages M2↑, M1/M2↓, TNF-α↓ IL-6↓, IL-1β↓, IL-10↑, renal fibrosis↓
Liu et al. 2017 [133]	uUC-MSC CM	UUO rat	Sham group, UUO group, UUO + CM group	Injected with hucMSC conditional medium (500 μL) via left renal artery after the surgery	GSH↑, ROS↓, MDA↓, α-SMA↓, TGF-β1↓, TNF-α↓, Col-I↓, E-cadherin↑, PCNA↑, cell apoptosis↓, renal interstitial fibrosis↓
Matsui et al. 2017 [116]	MSCs	UUO rat	Sham group, UUO group, sham plus MSCs group, and UUO plus MSCs group (6 animals/group)	1 × 10^6^ BM-MSCs through the renal artery	Col-I↓, Col-III↓, fibronectin↓, α-SMA↓, p-STAT3↓, MMP-9↓, TIMP-1↓, TIMP-1/MMP-9↑, inhibit renal fibrosis
Rodrigues et al. 2017 [134]	huMSC	IRI rat	Control group, n = 4; IRI group, n = 9; IRI + huMSC group, n = 5	Cells (1 × 10^5^) were intraperitoneally administered to rat	BUN↓, Scr↓, FENa↓, TGF-β1↓, HO-1↓, miR-29a↓, miR-34a↓, miR-29b→, miR-335→, inhibit renal fibrosis
Song et al. 2017 [23]	AMSCs	UUO rat	Sham group, UUO group, UUO + ADSCs group (n = 15)	Injected with ADSCs (5 × 10^6^) or vehicle via tail vein	MCP-1↓, TLR4↓, TNF-α↓, IL-1β↓, IL-6↓, TGF-β1↓, Smad2/3↓, Smad7↑, α-SMA↓, FSP-1↓, FN↓, E-cadherin↑, renal fibrosis↓, Scr→, BUN→
Zhu et al. 2017 [135]	AMSCs	IRI mice	Normal group, I/R group, I/R + MSCs group	Injected with MSCs via tail vein	α-SMA↓, PDGFR-β↓, Sox9↑, FN↓, Col-I↓, TGF-β1↓, p-Smad3/Smad3↓, IL-6↓, IL-10↑, IL-1β↓, TNF-α↓, FACS↓, renal fibrosis↓
Rota et al. 2018 [136]	uUC-MSC	ADR-induced nephropathic athymic rat	Control group, ADR group, ADR + BM-MSCs group, ADR + UC-MSCs group, ADR + kPSCs group, ADR + CM-UC-MSCs group	Intravenous infusion of MSCs	Glomerular podocyte and endothelial cell injury↓
Wu et al. 2018 [137]	WJ-MSCs-MV	Rat kidney transplant IRI model	Sham group (n = 40); kidney transplant IRI group (n = 40); MV-injected kidney transplant IRI group (n = 40)	100 μg MVs in 1 mL vehicle was administered via tail vein after kidney transplantation	Scr↓, BUN↓, vWF↓, IL-10↓, TNF-α↓, Ki67↑, cell apoptosis↓, α-SMA↓, TGF-β1↓, HGF↑, renal fibrosis↓
Zou et al. 2018 [138]	AMSCs	IRI mice	Sham group (n = 8), RAS + vehicle group (n = 10), RAS + AMSCs group (n = 10), and RAS + KIM-AMSCs group (n = 10)	Cells (5 × 10^5^) were injected through the carotid artery	BAX↓, CTGF↓, PAI→, TIMP1→, cell apoptosis↓, inhibit renal fibrosis

### Therapeutic effects of AMSCs on renal fibrosis

In 2001, Zuk et al. [139] successfully isolated MSCs from adipose tissue for the first time. In a transplantation study of autologous AMSCs, Song et al. [23] transplanted AMSCs into UUO model rats by tail vein injection and reported that AMSCs decreased MCP-1, TLR4, TNF-α, IL-1β, IL-6, TGF-β1, Smad2/3, α-SMA, fibroblast-specific protein 1 (FSP-1), and FN and inhibited renal fibrosis. However, AMSCs could increase Smad7 and E-cadherin, but Scr and BUN were not changed. Burgos-Silva et al. [128] injected AMSCs into FVB mice by intraperitoneal injection to compare the efficacy of AMSCs on acute kidney injury (AKI) and CKD. The results showed that AMSCs decreased CXCL1, CCL-5, MPO, and PCNA, but MCP-1, IL-2, IL-6, GM-CSF, MIP-1a, and BUN were not changed. Donizetti-Oliveira et al. [117] conducted a study in a mouse model induced by ischemia reperfusion injury (IRI), and treatment with allogeneic AMSCs reduced IL-1α, IL-6, TNF-α, IL-4, IL-12, RANTES, FSP-1, Col-I, and Hypoxyprobe and inhibited renal fibrosis. However, the expression of IL-10 and HO-1 was increased by AMSCs. Furthermore, KC, IL-1β, and IL-13 were not changed. Quimby et al. [122] injected AMSCs intravenously in a cat CKD model and showed that AMSCs can did not change Scr, BUN, IL-8, MCP-1, TGF-β1, or VEGF. Zou et al. [138] injected AMSCs through the carotid artery in IRI mice, and the results showed that AMSCs could decrease BAX and CTGF and inhibit apoptosis and renal fibrosis, but the expression of PAI and TIMP1 was not changed. A similar cat study conducted by the same team showed no significant changes in BUN, serum creatinine, phosphorus, potassium, the glomerular filtration rate (GFR), urinary albumin/creatinine ratio (UACR), or the volume of filled cells in cats injected with allogeneic AMSCs [140].

In a study of xenogeneic AMSC transplantation, Katsuno et al. [121] cultured human AMSCs with low serum and high serum, and these AMSCs and high-serum AMSCs were injected into an AKI rat model induced by folic acid. The results showed that the degree of mesenchymal fibrosis in the low-serum adipose mesenchymal stem cell group was lower than that in the high-serum adipose mesenchymal stem cell group. However, AMSCs did not change Scr, BUN, HGF, or VEGF levels. In addition, Zhu et al. [135] injected human AMSCs into an IRI rat model, and the results showed that AMSCs decreased α-SMA, PDGFR-β, FN, Col-I, TGF-β1, p-Smad3/Smad3, IL-6, IL-1β, TNF-α, and FACS and inhibited renal fibrosis, but increased Sox9 and IL-10. Iwai et al. [126] injected AMSCs systemically via the penile vein or via the renal artery in a rat DCD renal transplantation model and showed MSCs could decrease Scr, BUN, and renal fibrosis and increase rat survival. Eirin et al. [132] injected AMSCs via the renal artery in an RAS pig model. The results showed that AMSCs decreased Scr, M1 macrophages, M1/M2 macrophages, TNF-α, IL-6, IL-1β, and renal fibrosis and increased IL-10 and the number of M2 macrophages (Table 1).

### Therapeutic effects of WJ-MSCs on renal fibrosis

The function of WJ-MSCs in the treatment of renal fibrosis has been especially studied. The extraction of WJ-MSCs can be carried out in the intervascular, perivascular, and subamniotic areas of Wharton’s jelly [141]. Du et al. [22] conducted a study in an IRI rat model with intravenous infusion of WJ-MSCs and showed that WJ-MSCs decreased the total renal collagen concentration, α-SMA levels, and renal fibrosis and increased E-cadherin and HGF/TGF. Du et al [119] also assessed the effects of WJ-MSCs on acute and chronic kidney injury induced by IRI and reported that WJ-MSCs inhibited Scr, BUN, collagen, α-SMA, renal fibrosis, and renal tubular cell apoptosis and increased p-Akt, HGF, HO-1, IL-10, and renal tubular cell proliferation.

Furthermore, Zhang et al. [127] injected human WJ-MSCs-MVs into IRI rats and found that MVs could eliminate IRI-induced fibrosis and improve renal function. Furthermore, the results showed that WJ-MSCs-MVs decreased Scr, BUN, NOX2, α-SMA, MDA, ROS, and Ki67 and alleviated apoptosis. Wu et al. [137] injected WJ-MSCs-MVs via the tail vein after kidney transplantation in a rat kidney transplant IRI model, and the results showed WJ-MSCs-MVs reduced the expression levels of Scr, BUN, vWF, IL-10, TNF-α, α-SMA, and TGF-β1, inhibited cell apoptosis and renal fibrosis, and increased Ki67 and HGF. As mentioned previously, human WJ-MSC-MVs can significantly reduce renal fibrosis (Table 1).

### Therapeutic effects of AF-MSCs on renal fibrosis

Sedrakyan et al. [118] established an animal model of Alport syndrome for the intracardiac transplantation of homogenetic AF-MSCs. The results indicated that AF-MSCs inhibited the progression of interstitial fibrosis and glomerulosclerosis before the onset of proteinuria. M2 macrophage polarization was also found in the kidneys of a mouse model. After kidney transplantation, autologous AF-MSCs were injected directly into the transplanted kidney via the renal artery. The results showed that the injection of AF-MSCs increased Col4a1, Col4a2, Col4a3, Col4a4, and Col4a5, but Col4a6 was not changed. Sun et al. [123] injected AF-MSCs via the tail vein in a UUO mouse model and showed that AF-MSCs decreased HIF-1α, TGF-β1, Col-I, MCP-1, Ki67, apoptosis, and renal fibrosis and increased VEGF, E-cadherin, and PCNA. Baulier et al. [125] showed that AF-MSCs can treat renal fibrosis in animal models, and this effect may be associated with AF-MSC-mediated enhancement of glomerular and tubular function through the TGF-β/Smad3 pathway, effectively improving renal fibrosis. The researchers also showed that AF-MSCs could decrease Scr, proteinuria, α-SMA, VEGF-A, Ang 1, and Flt-1. Monteiro Carvalho Mori da Cunha et al. [129] injected AF-MSCs into the renal artery in an IRI rat model, and the results showed that AF-MSCs decreased Ki67, α-SMA, CD68, tubular necrosis, tubular hyaline casts, and renal fibrosis, but Scr was not changed (Table 1).

### Therapeutic effects of mesenchymal stem cells from other sources on renal fibrosis

Huang et al. [120] injected UC-MSCs via the tail vein in a UUO rat model, and the results showed that MSCs inhibited renal fibrosis. Zhou et al. [124] injected UC-MSC-derived exosomes into a cisplatin-induced AKI rat model, and the results showed that UC-MSC-derived exosomes decreased 8-OhdG, MDA, and caspase 3 and increased GSH. Zou et al. [131] injected UC-MSCs-MVs via the caudal vein after IRI, and the results showed that hu-MSCs decreased Scr, BUN, apoptosis, HIF-1α, α-SMA, and renal fibrosis and increased Ki67, VEGF, PHD2, and VHL. Liu et al. [133] injected UC-MSC-conditioned medium (CM) (500 μL) via the left renal artery after surgery in UUO rats, and the results showed that UC-MSC-CM decreased ROS, MDA, α-SMA, TGF-β1, TNF-α, Col-I, apoptosis, and renal interstitial fibrosis and increased GSH, E-cadherin, and PCNA. Rota et al. [136] intravenously infused UC-MSCs in an ADR-induced nephropathic athymic rat model and showed that UC-MSCs weakened glomerular podocyte and endothelial cell injury. Rodrigues et al. [134] injected hu-MSCs via vein injection in an IRI rat model, and the results showed that hu-MSCs could decrease BUN, Scr, FENa, TGF-β1, HO-1, miR-29a, miR-34a, and renal fibrosis, but miR-29b and miR-335 were not changed. DMSCs are MSC-like cells that are present in the human body for a lifetime [142]. Studies have shown that cryopreservation of DMSCs can promote the regeneration of renal tubule structure in rats with acute renal failure. In addition, Hattori et al. [130] confirmed that DMSCs could reduce inflammatory cytokines and promote renal function in acute renal injury caused by ischemia reperfusion injury. The researchers showed that DMSCs reduced Scr, BUN, MIP-2, IL-1β, and MCP-1 and alleviated renal macrophage and neutrophil infiltration (Table 1).

## Clinical applications of MSCs in the treatment of renal fibrosis (safety and efficacy)

As mentioned previously, in most preclinical trials, BM-MSCs induced almost no adverse reactions and are considered safe, but their efficacy is still controversial [122, 140, 143]. However, for human translation, safety is a serious concern and an obstacle to the smooth transition from the laboratory. What is concerning is that patients are vulnerable to infection because of their physical condition and technical problems. Immunomodulatory effects, potential carcinogenicity, the active proliferation of stem cells, cell embolism, the acute and chronic immunogenicity of cells, and zoonosis induced by cell culture reagents [144]. A clinical phase I trial showed that after cardiac surgery, patients with a high risk of AKI received allogeneic MSCs, and allogeneic MSCs were found to be feasible and safe [145]. Tan et al. [146] found that, compared with anti-IL-2 receptor antibody induction therapy, autologous BM-MSCs could reduce the risk of opportunistic infection, reduce the incidence of acute renal rejection, and better predict renal function. Furthermore, BM-MSCs did not increase adverse events and had no negative impact on the survival rate of renal transplantation. Reinders et al. [147] extracted BM-MSCs from patients with end-stage renal disease (ESRD) and age-matched healthy controls and found that these cells had similar phenotypic and functional characteristics. In addition, the researchers also evaluated the effect of autologous BM-MSCs on rejection after kidney transplantation and found that renal interstitial fibrosis/tubular atrophy (IF/TA) was alleviated in 2 patients with renal tubulitis without rejection, and the use of autologous BM-MSCs in transplantation, IF/TA, and subclinical rejection are clinically practical and safe [148]. The researchers also showed that autologous BM-MSCs in combination with the mTOR inhibitor everolimus could promote tacrolimus withdrawal and reduce renal fibrosis in renal transplant recipients. Good results were obtained for BM-MSCs in the prevention of renal fibrosis, which were shown to be safe and protected kidney structure and function, suggesting that patients on waiting lists for renal transplantation have made good progress [149]. We worried about allogeneic immune responses to donor-derived cell therapy in patients with renal transplantation. Allogeneic MSCs could elicit an immune response against the donor, which would increase the incidence of organ rejection and impact the survival of allografts. Reinders et al. [150] tried to use allogeneic BM-MSCs to confirm their efficacy and safety in renal transplant recipients, and they showed that allogeneic BM-MSCs are an effective and safe treatment. In our previous study [151], we found that in patients with systemic lupus erythematosus, the MSC group had lower proteinuria than the control group, and the MSC group also exhibited a lower rate of adverse events than the control group.

However, a study indicated that MSC treatment might not achieve good efficacy in clinical studies. Kim et al. [152] examined a patient with CKD to assess the efficacy of autologous AMSCs. The patient’s renal function was stable for many years before AMSC administration. The preexisting renal insufficiency was rapidly exacerbated 1 week after the autologous MSC infusion, and the renal biopsy showed inflammatory cell infiltration and severe renal interstitial fibrosis, with a few stem cells. The results showed the potential nephrotoxicity of autologous AMSC therapy in CKD patients.

As mentioned previously, at present, there have been relatively few clinical studies on stem cells in the treatment of renal fibrosis. Additional studies should be conducted to assess the safety and efficacy of MSCs in the treatment of renal fibrosis.

## Conclusion

The occurrence of renal fibrosis may be associated with the TGF-β/Smad, Notch, Hedgehog, Wnt, TNF-α, NF-κB, MAPK, JAK/STAT, PI3K/AKT, and RHO/ROCK signaling pathways. To date, some papers have indicated that stem cells can regulate the TGF-β/Smad, NF-κB, MAPK/ERK, PI3K/AKT, and TNF-α signaling pathways to alleviate renal fibrosis. No study has shown that stem cells regulate the Notch, Hedgehog, Wnt, RHO/ROCK, or JAK/STAT signaling pathways to treat renal fibrosis. Further studies are needed to confirm that stem cells can act through the Notch, Hedgehog, Wnt, JAK/STAT, and RHO/ROCK signaling pathways to treat renal fibrosis.

## Discussion

At present, the idea of using stem cells in the treatment of renal fibrosis has mainly focused on the mechanism of fibrosis for the purpose of treating renal fibrosis by inhibiting fibrotic signaling pathways. Compared with other treatments for renal fibrosis, stem cells have several advantages.

1. Stem cells have strong proliferation abilities and multidirectional differentiation potential and can proliferate, differentiate, and produce various progeny.
2. Inhibiting of T cell proliferation and the immune response through intercellular interactions and the production of cytokines modulates the immune response.
3. Stem cells are convenient and easy to isolate, culture, amplify, and purify, and these cells retain the characteristics of stem cells after repeated passages of amplification.
4. Stem cells have low immunogenicity. Since these cells are in a primitive state, they are not easily recognized, and so there is no problem regarding blood type matching and no characteristics of immune rejection.
5. Long-term passage can be used to differentiate these cells into various cell types, such as renal innate cells, muscle cells, liver cells, osteoblasts, and chondrocytes, without changing their biological characteristics.
6. Homing (targeting -- targeting) is a characteristic of these cells. Damage signals can stimulate the migration and differentiation of stem cells to damaged organs and tissues, facilitating homing to damaged lesions and the repair of damaged cells.

However, there are also challenges to the use of stem cell therapy for renal interstitial fibrosis.

1. The mechanism by which stem cells reduce renal interstitial fibrosis is limited to conclusions drawn from experimental evidence, and no unified specific theory has been developed.
2. Most research in the field of stem cells in the kidney has been limited to animal or in vitro experiments, the source of stem cells and their purification are not standardized, and there have been few reports of real stem cells used in the clinical treatment of renal fibrosis. There is a lack of standardized renal disease treatment in the clinic. Specific stem cells used for the clinical treatment of renal fibrosis are consistent with animal models, and the conclusions still need clinical trials to be confirmed.
3. The differences in the methods used to obtain stem cells cannot be ignored, and rigorous protocols for the characterization, handling, and delivery of stem cells are lacking. The stem cell culture medium cannot be cleaned completely. After entering the human body, this medium may induce allergic reactions
4. Stem cells have the property of multidifferentiation, and if applied in clinical practice, it is possible for exogenous stem cells to differentiate into nontarget products such as tumors and fat after implantation in vivo. The safety of transplanted stem cells has not been clearly examined, and additional clinical trials are needed to draw conclusions.

## Expectation

To date, although no study has confirmed that stem cells can alleviate renal fibrosis through the Notch, Hedgehog, Wnt, Rho/Rock, or JAK/STAT signaling pathways, we believe that these mechanisms will be confirmed through continuous experimental studies. If experimental data show that stem cells are effective in the treatment of renal fibrosis, then the clinical application of stem cells will be a new option for the treatment of renal fibrosis.

## Data Availability

Data sharing is not applicable to this article, as no datasets were generated or analyzed during the current study.

## Abbreviations

BM-MSCs: Bone marrow mesenchymal stem cells
UC-MSCs: Umbilical cord blood mesenchymal stem cells
AF-MSCs: Amniotic fluid mesenchymal stem cells
AMSCs: Adipose mesenchymal stem cells
WJ-MSCs: Wharton’s jelly-derived MSCs
DMSCs: Dental mesenchymal stem cells
ECM: Extracellular matrix
CKD: Chronic kidney disease
EMT: Epithelial to mesenchymal transformation
TIMPs: Tissue inhibitor of metalloproteinases
MMPs: Matrix metalloproteinases
PI3K/AKT: Phosphatidylinositol-3 kinase
TGF-β1: Transforming growth factor-β1
NF-κB: Nuclear factor-κB
PTCH1: Patched1
GSK3B: Glycogen synthase kinase 3B
CK1: Casein kinase 1
PKA: Protein kinase A
Smo: Smoothened
Gli: Zinc finger protein
CK1α: Casein kinase 1α
Rock: Rho coil kinase
JAK-STAT: Transcription/signal transducers and activators of transcription
MAPK: Mitogen-activated protein kinase
TNF-α: Tumor necrosis factor α
SP1: Sphingosine 1-phosphate
PTEN: Phosphatase and tensin homolog
MMT: Macrophage-myofibroblast transition
EndoMT: Endothelial-mesenchymal transformation
α-SMA: α-Smooth muscle actin
PAI-1: Plasminogen activator inhibitor-1
PCNA: Proliferating cell nuclear antigen
LPS: Lipopolysaccharide
IFN-γ: Interferon-γ
ROS: Reactive oxygen species
HGF: Hepatocyte growth factor
MFG-E8: Milk globule epidermal growth factor 8
GFR: Glomerular filtration rate
UACR: Urinary albumin/creatinine ratio
AKI: Acute kidney injury
FSP-1: Fibroblast-specific protein 1
CBP: CREB-binding protein
Shh: Sonic hedgehog
Ihh: Indian hedgehog
Dhh: Desert hedgehog
UUO: Unilateral ureteral obstruction
IRI: Ischemia reperfusion injury
CM: Conditioned medium
IF/TA: Renal interstitial fibrosis/tubular atrophy
NICD: Notch intracellular domain
